# Dietary preferences affect the gut microbiota of three snake species (Squamata: Colubridae)

**DOI:** 10.3389/fmicb.2025.1559646

**Published:** 2025-05-21

**Authors:** Guangxiang Zhu, Huina Song, Mingwen Duan, Ji Wang, Jingxue Luo, Shijun Yang, Fei Wu, Jiuyan Jiang, Ji Chen, Wenjiao Tang

**Affiliations:** College of Life Science, Sichuan Agricultural University, Ya’an, China

**Keywords:** gut microbiota, dietary preference, Snake, 16S r RNA, evolution

## Abstract

**Introduction:**

The gut microbiota is an emerging frontier in animal research, and researchers are increasingly transparent about its importance to animal health. Reptiles, particularly snakes, have not received the same attention given to other vertebrates, and the composition of their wild gut microbiome remains understudied.

**Methods:**

In this study, the HiSeq high-throughput sequencing platform was used to sequence and analyze the 16S rRNA V4 region of the gut microbiota of three species (*Gonyosoma coeruleum*, *Rhabdophis pentasupralabralis*, *Rhabdophis tigrinus*).

**Results:**

This study investigated alpha diversity analysis and showed that the gut microbiota richness of RP was significantly higher than that of the other two snakes. The dominant genus of *Gonyosoma coeruleum* (GC) and *Rhabdophis tigrinus* (RT) is *Cetobacterium*, while Enterobacteriaceae; *g_uncultured* is the dominant genus of *Rhabdophis pentasupralabralis* (RP). Tree clustering based on Bray-Curtis distances and Jaccard similarity coefficients indicated that the gut microbiota composition of *RP* and *RT* was more similar. The unique diet of RP promotes a diverse, competitive gut microbiota, while GC and RT displayed more stable networks linked to shared dietary habits. The functional heat map showed that the predicted functions of the gut microbes of the three snake species were different. These findings suggest that dietary preferences exert a stronger influence on gut microbial composition and function than host genetic background, and distantly related species with similar diets exhibit convergent gut microbiota characteristics.

## 1 Introduction

The intestinal tract of animals has a complex and diverse bacterial community whose functions vary from nutrient absorption to disease regulation, even influencing animals’ daily behavior and speciation (Dominguez-Bello et al., 2019). Simultaneously, gut microbiota composition is influenced by various factors, including species diversity, dietary patterns, environmental conditions, seasonal variations, and ecological niches (Xiao et al., 2022). Complex interactions exist between intestinal microorganisms, their metabolites, and host cells. Among them, short-chain fatty acids are the most crucial bacterial metabolites as they serve as direct energy sources for host cells, thereby stimulating the production of intestinal hormones and regulating food intake in the brain (Cani et al., 2019). Moreover, the high-protein diet of carnivores can enhance amino acid metabolism by intestinal microbes, leading to short-chain fatty acid (SCFA) production. This process may support intestinal barrier function, thereby aiding the host in adapting to a high-protein diet (Neis et al., 2015). Snakes, as quintessential carnivorous reptiles, possess protein-rich, carbohydrate-poor diets, along with a distinctive intermittent feeding pattern. Their digestive systems adhere to Krogh’s principle of comparative physiology, enabling highly efficient nutrient absorption (Glaudas et al., 2019). For instance, in *Python bivittatus* digestion, Firmicutes abundance and diversity exhibit significant increases (Costello et al., 2010). Energy metabolism-related enzymes produced by Fusobacteria, Bacteroidetes, and Firmicutes contribute to the degradation of various macromolecules in food, playing a pivotal role in nutrient availability for the host.

Recent studies have revealed that dietary habits and host genetics are the primary factors shaping reptile gut microbiota, exerting a significantly greater influence than environmental variables or conservation status. Reptilian gut microbiota exhibits relatively stable core components, predominantly composed of Bacteroidetes, Proteobacteria, and Firmicutes (Hoffbeck et al., 2023). Multiple studies indicate that host genetic background, whether through maternal transmission or genotype-environment interactions, profoundly influences microbial community structure (Goodrich et al., 2014). For instance, comparisons of farm-raised *Naja atra*, *Ptyas mucosa*, *Elaphe carinata*, and *Deinagkistrodon acutus* fed either chicken or mice have demonstrated that host species significantly shape the taxonomic composition and diversity of gut microbiota (Zhang et al., 2019). However, cross-species analyses suggest that dietary heterogeneity exerts a stronger influence on microbiota composition than phylogenetic relatedness (Barelli et al., 2015). A global meta-analysis of 113 vertebrate species further highlighted a strong correlation between host dietary patterns and gut microbiome variation (Xie et al., 2024). Dietary factors drive the adaptive evolution of microbial communities through selective pressures, with the host diet potentially playing a more dominant role than phylogenetic relationships (Jiang et al., 2017). Comparative studies have shown that, relative to herbivorous turtles, *Caretta caretta* shares more gut microbiota features with carnivorous marine mammals (Biagi et al., 2019). Additionally, *Gekko japonicus* exhibits significant differences in gut microbiota diversity between captive and wild populations, further implicating diet as a key determinant of microbial composition (Jiang et al., 2023). However, our understanding of differences in the composition and function of gut microbes in wild snakes caused by host genetics and dietary preferences is still limited, but it is of great significance for biodiversity conservation.

**Figure 1:**
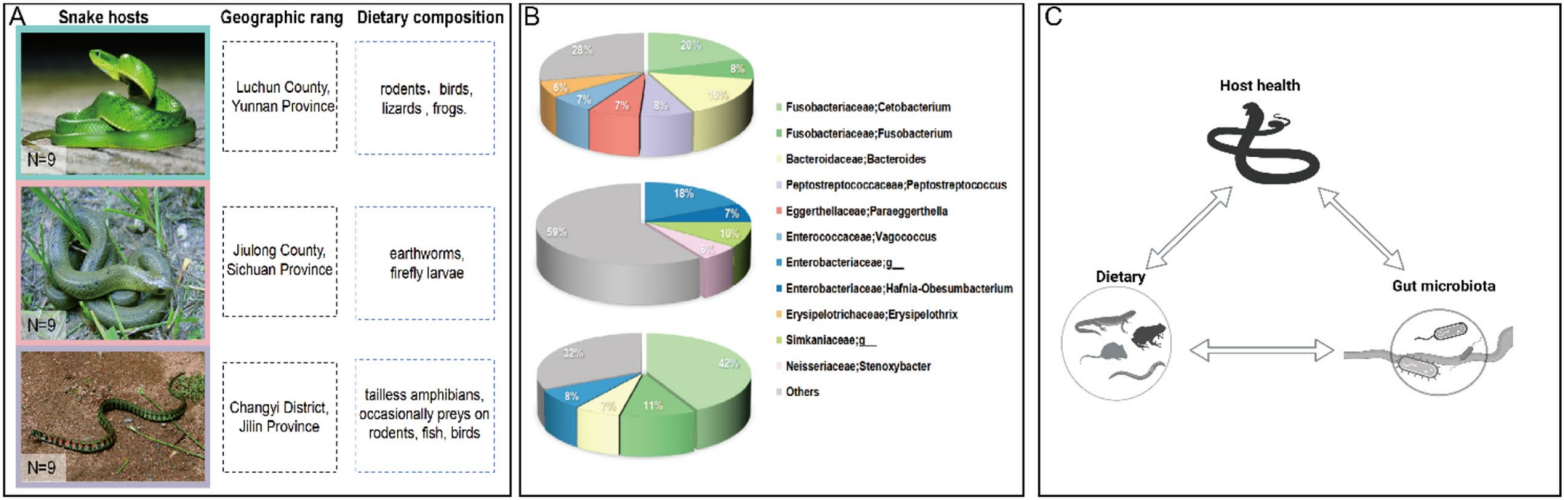
**(A)** Color-coded photographs of snake species, including GC framed in teal, RP framed in pink, and RT framed in light purple, alongside their respective study sites and dietary compositions (photographs courtesy of Fanyii Lai and our team). **(B)** Pie charts illustrate the composition of the snake gut microbiome at the genus level. Each color corresponds to specific taxonomic groups: for instance, green represents Fusobacteria, with different green shades denoting various genera within Fusobacteria (“Others” aggregates all microbial genera that did not meet the 5% threshold). (C) A Conceptual Model of Diet-Microbiota-Host Interactions in Snakes was created with BioRender (Song, 2025).

In the present study, we conducted an in-depth analysis of three snake species: *Gonyosoma coeruleum* (GC)*, Rhabdophis pentasupralabialis* (RP), and *Rhabdophis tigrinus* (RT)*. Gonyosoma coeruleum,* first described in 2021 (Liu et al., 2021), predominantly inhabits China (Yunnan Province), Vietnam, Thailand, western Malaysia, and southeastern Myanmar, and in 2022, it was thought to be equally distributed in China (Sichuan, Guizhou, and Hainan) (David et al., 2022). It usually inhabits forests in hills and low mountains and feeds mainly on rodents, birds, lizards, and frogs. *Rhabdophis pentasupralabialis* was named in 1983 (Zhao, 1995). It is a species-group nuchalis, genus *Rhabdophis*, mainly distributed in China (Sichuan Province; Jiulong City, Yunnan), feeding on earthworms and firefly larvae (Yoshida et al., 2020). *Rhabdophis tigrinus* is a wide-ranging species of the genus *Rhabdophis*, with a preference for moist areas, mainly in mountainous, hilly, and plain areas near water, but also far away from water. It prefers wet areas and mainly inhabits near water in mountainous, hilly, and plain areas, but is also found in wet and grassy mountainous areas far from water, mainly feeding on tailless amphibians, occasionally preying on rodents, fish, and birds (He and Yu, 2007; Figure 1A). RT and GC are generalist feeders, preying on vertebrates, whereas RP is a specialist feeder, restricted to invertebrates. All three species belong to the family Colubridae, with RP and RT classified under the genus *Rhabdophis*, while GC belongs to the genus *Gonyosoma*. In the phylogenetic tree constructed from seven nuclear loci and five mitochondrial genes, RP and RT are on the same branch, while GC is more distant (Pyron et al., 2013).

We hypothesize that: (1) Dietary diversity is positively correlated with microbiota *α*-diversity; (2) Specific prey types promote the enrichment of distinct functional microbial communities; (3) The genetic background of the host establishes the foundational structure of the gut microbiota, upon which diet further modulates microbial function and composition, with both factors collectively determining the final gut microbiota composition in snakes.

## 2 Materials and methods

### 2.1 Sample collection and DNA extraction

A total of nine adult male snakes, three for *Gonyosoma coeruleum* (Colubridae, Serpentes) were captured from Lvchun county, Yunnan province, China, in July 2015; three for *Rhabdophis pentasupralabralis* (Colubridae, Serpentes) were captured from Jiulong County, Sichuan province, China, in June 2015; and three for *Rhabdophis tigrinus* (Colubridae, Serpentes) were captured from Changyi District, Jilin province, China, in August 2015 (Figure 1A). The snake sampling procedure was approved by the Institutional Animal Care and Use Committee of the Sichuan Agricultural University (Approval No.20150036). Snakes were transported to the Sichuan Agricultural University laboratory for sampling without feeding during transportation. Before sampling, snakes were manually palpated to confirm no prey remained in the gastrointestinal tract. Aseptic dissection was performed to collect contents from the large intestine, small intestine, and cloaca of each individual. Samples from these three regions were pooled, resulting in 9 composite samples per snake (total *n* = 27). Specimens were immediately placed into 2 mL sterile collection tubes, snap-frozen in liquid nitrogen, and stored at −80°C. Genomic DNA from the gut tissues was extracted by using a TIANamp Stool DNA Kit (Tiangen Biotech, Beijing). DNA integrity was confirmed by observing high molecular weight bands (>10 kb) on agarose gels, while purity (A260/A280 = 1.8–2.0) and concentration were quantified using a NanoDrop 3,300 (Thermo Scientific). Microbial DNA stability under prolonged −80°C storage has been previously validated (Gavriliuc et al., 2021; Vandeputte et al., 2017), mitigating concerns about degradation in samples collected in 2015.

### 2.2 16S rRNA gene amplification

The V4 hypervariable region of the 16S rRNA gene was amplified with primers 515F/806R (Suzuki and Nachman, 2016), using a 30 μL reaction containing Phusion^®^ High-Fidelity PCR Master Mix (New England Biolabs), primers (2 μm), and template DNA (10 ng). Cycling: 98°C 1 min; 30 × [98°C 10 s, 50°C 30 s, 72°C 30 s]; 72°C 5 min. Products were verified by 2% agarose gel. Amplicon Sequence Variant (ASV) was generated to classify taxa, replacing traditional Operational Taxonomic Units (OTUs). ASVs resolve single-nucleotide differences, minimize spurious taxa clustering, and enhance reproducibility compared to OTUs’ reliance on arbitrary 97% similarity thresholds. By leveraging ASV, this approach strengthens ecological inference capabilities for complex microbial communities (Callahan et al., 2017).

### 2.3 Illumina HiSeq platform sequencing

Amplicons (400–450 bp) were gel-purified (Thermo Scientific GeneJET Kit), normalized, and used to construct libraries with the TruSeq DNA PCR-Free Kit (Illumina). Library quality was verified via Qubit^®^ 2.0 (Thermo Scientific) and Agilent Bioanalyzer 2100. Finally, the libraries were sequenced on an Illumina HiSeq2500 platform, generating 250 bp paired-end reads at Novogene (Beijing, China).

### 2.4 Statistical analysis

Paired-end reads were demultiplexed using sample-specific barcodes, followed by trimming of barcode and primer sequences with Cutadapt v4.0 (Hall and Beiko, 2018). Sequences were merged via FLASH v1.2.11 (Magoc and Salzberg, 2011), and quality filtering was conducted in QIIME2 to generate clean tags. ASV was resolved through DADA2 (Callahan et al., 2016), which implemented rigorous quality control, corrected amplicon errors, and denoised sequences. Chimeric sequences were identified and removed by alignment against the SILVA 132 reference database. Taxonomic annotation employed the Silva 132 database, and the generated BIOM format ASV table is used for more complex data analysis downstream.

The alpha and beta diversity analyses based on ASV tables compared microbial composition and abundance among the three snake species. Beta diversity was quantified using Bray–Curtis distance matrices. Due to microbial community data’s high-dimensional and non-normally distributed nature, nonparametric permutational multivariate analysis of variance (PERMANOVA) was employed as the primary method. In addition, PERMDISP tests were conducted to account for the potential confounding effects of intragroup dispersion on PERMANOVA results. Pairwise comparisons were carried out using ANOSIM with 999 permutations and Benjamini–Hochberg correction for multiple testing, providing quantitative assessments of intergroup differences. All analyses were performed in R, and visualizations were generated using the ggplot2 package (Tang et al., 2016). Phylogenetic trees were reconstructed using the Maximum Likelihood (ML) method based on mitochondrial cytochrome b (*cytb*) gene sequences (the sequences of RT and RP were measured by our group) from the three snake species (Das, 2006). Individual-level phylogenetic distance matrices were computed using MEGA7 (Kumar et al., 2016). Correlations between host phylogenetic distances and microbial community distances were evaluated via Mantel tests (999 permutations). Alpha diversity and beta diversity were calculated and visualized using R software.

To compare differences in microbiota abundance, the top 10 phyla and top 20 genus abundances were obtained and visualized using bar charts. Differences in the relative abundance of phyla and genera were expressed as mean ± SD, and one-way ANOVA (LSD test, *p* < 0.05) was used to compare the gut microbiota of different snakes. Significant differences in all genera were also analyzed using online LEfSe (LDA > 4). Membership and structure of samples at ASV were revealed by Venn diagrams and PCoA plots. Microbial functions were predicted using PICRUSt2 v2.3.0 (Douglas et al., 2020) via ASV phylogenetic placement. KEGG pathway predictions were retained only if NSTI scores <0.15. STAMP was used to analyze differential KEGG pathways between groups via two-sided Welch’s *t*-test (*p* < 0.05). Venn diagram analysis and visualization were performed using the online platform EVenn (Yang et al., 2024). Linear discriminant analysis (LDA) effect size (Lefse), and histograms were plotted using BioScience Cloud^1^ (Gao et al., 2024). For the gut bacterial network, we determined significant associations for genera with relative abundance >1% for each species by R4.3.1 using the Spearman correlation test (Bastian et al., 2009). The heatmap of predicted function analysis was performed using the OmicShare tools, a free online platform for data analysis.^2^

## 3 Results

### 3.1 Sequencing data quality of snakes

Following quality control, we obtained 788,913 high-quality reads from 27 intestinal samples. Samples averaged 29,219 reads, generating 4,012 amplicon sequence variants (ASVs). Taxonomic classification identified 35 phyla, 101 classes, 275 orders, 522 families, and 1,127 genera across the three wild snake species. Good’s coverage index exceeded 99.7% for all samples, indicating >99% bacterial community identification (Table 1).

**Table 1:** Alpha diversity analyses of bacterial 16S rRNA gene high throughput sequencing data.

	Observed_species	Chao1	ACE	Shannon	Simpson	Goods_covers
GC	206.11 ± 44.14	210.28 ± 44.94	210.60 ± 44.44	2.82 ± 0.63	0.82 ± 0.12	0.999 ± 0.0002
RP	410.67 ± 244.73	417.99 ± 252.03	417.72 ± 251.74	3.44 ± 0.96	0.84 ± 0.15	0.999 ± 0.0008
RT	234.67 ± 107.23	239.39 ± 107.28	240.40 ± 108.14	2.81 ± 0.69	0.79 ± 0.14	0.999 ± 0.0004
P	0.009	0.011	0.011	0.21	0.518	0.879

Data are expressed as Mean ± SD, *P* ≤ 0.05 indicates a significant difference.

### 3.2 Comparison of gut microbiota diversity and clustering differences

**Figure 2:**
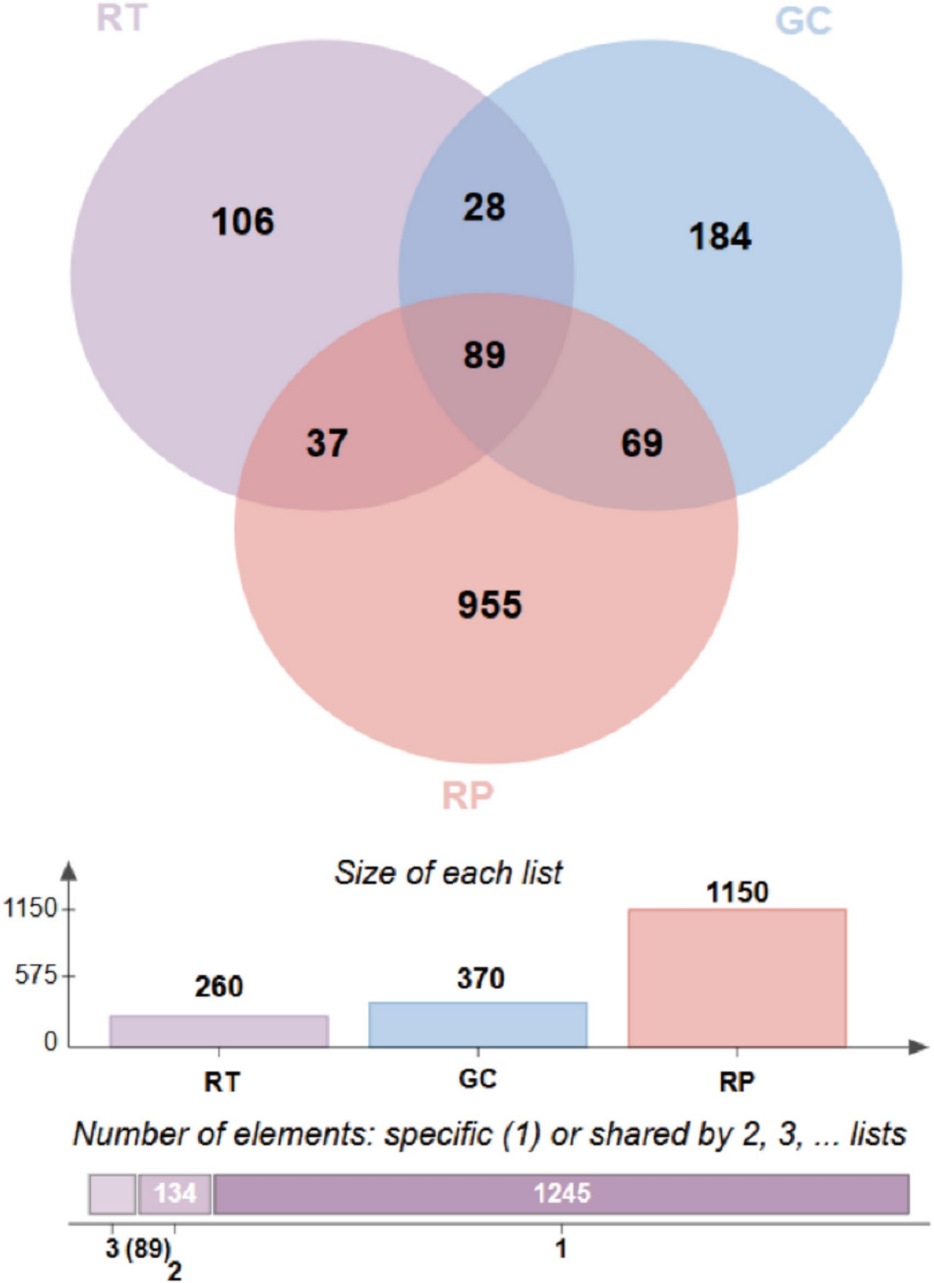
The Venn diagram of shared ASV among microbiota in different groups.

From Supplementary Figure S1 and Table 1, Observed_species (410.67 ± 244.73), Chao1 (417.99 ± 252.03), and ACE (417.72 ± 251.74) index of intestinal microorganisms in RP were significantly higher than GC (206.11 ± 44.14, 210.28 ± 44.94, 210.60 ± 44.44) and RT (234.67 ± 107.23, 239.39 ± 107.28, 240.40 ± 108.14). Venn analysis revealed 184 unique ASVs in GC compared to 106 in RT. In particular, the number of unique ASVs (955) in RP was significantly higher than in GC and RT. The number of ASVs shared by all snakes is 89 (Figure 2).

**Figure 3:**
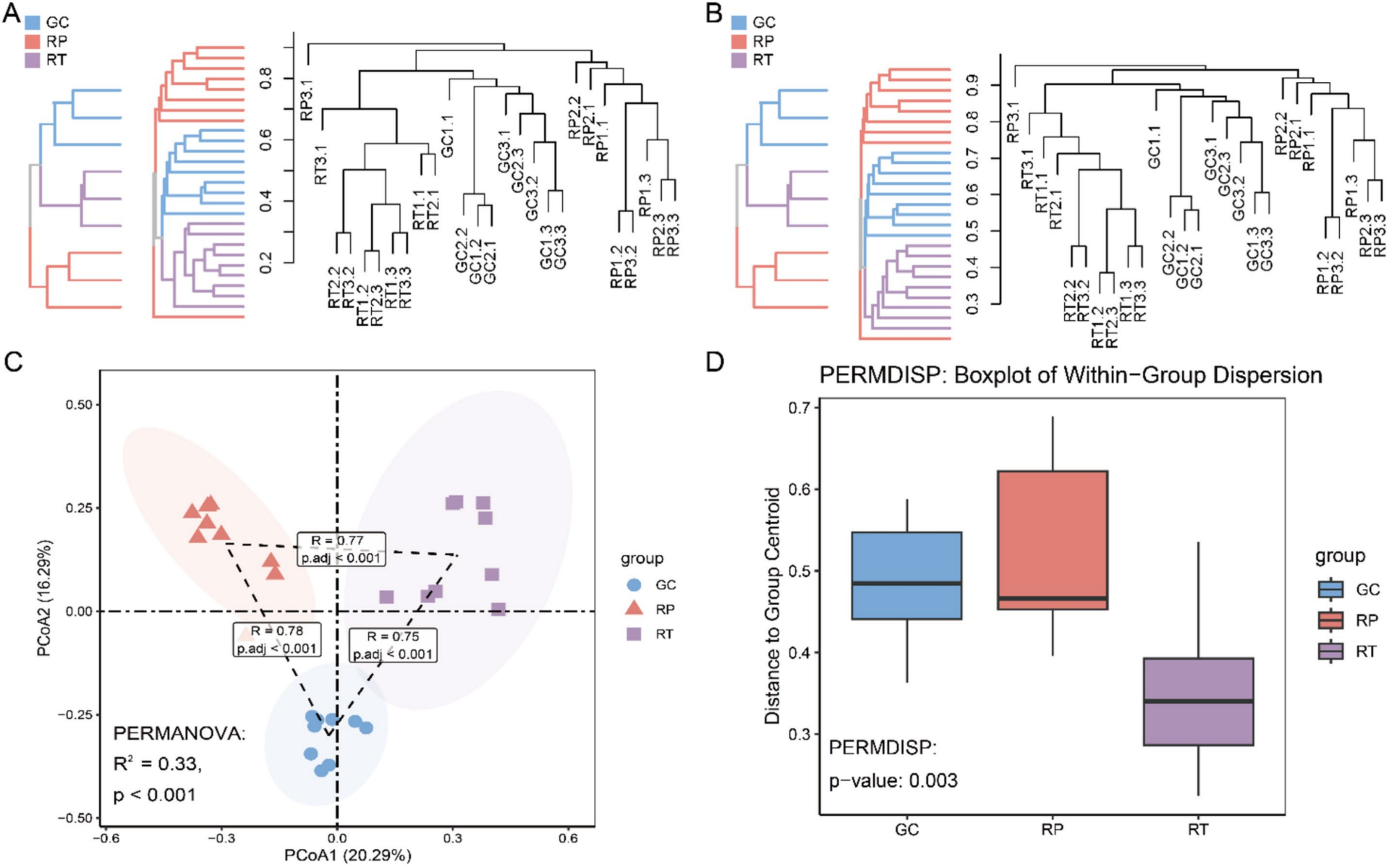
Based on the ASV abundance, UPGMA tree clustering based on **(A)** Bray-Curtis distance and **(B)** Jaccard similarity coefficient. **(C)** PCoA (Principal Coordinates Analysis) of the ASV abundance table, performed with Bray-Curtis distance and a 95% confidence interval. Adonis (PERMANOVA, permutational multivariate analysis of variance) was utilized to assess whether significant distribution differences existed among the three sample groups, supported by pairwise comparisons via ANOSIM. **(D)** Box plots displaying the outcomes of PERMDISP tests, which evaluate inter-group dispersion differences.

To explore the microbial community structure and similarity of different samples, we employed the Bray-Curtis distance and Jaccard similarity coefficients for clustering analyses. Our results showed that the branches of GC and RT showed some clustering in the graph and were separated from RP, possibly reflecting the similarity between GC and RT in terms of colony composition. The RP population was independent as a bifurcation, indicating that the structure of their microbial community was different from that of GC and RT (Figure 3A; Figure 3B). Principal coordinate analysis (PCoA) showed the first two principal coordinates explained 20.29 and 16.29% of the variance. The PCoA plot (Figure 3C) demonstrated significantly greater intragroup similarity compared to interspecific differences, showing clear interspecific clustering patterns. At the 95% confidence level, GC and RT exhibited more similar microbiota profiles than RP. PERMANOVA analysis confirmed significant overall compositional differences among groups (*R*^2^ = 0.33; *p* < 0.001), while PERMDISP (Figure 3D) indicated significant intragroup dispersion heterogeneity (*p* < 0.05), potentially reflecting individual physiological variation or microenvironmental dynamics. To account for dispersion effects and quantify intergroup differences, pairwise ANOSIM comparisons confirmed significant compositional differences (Figure 3C) for all comparisons (adjusted *p* < 0.001) with high R values (GC vs. RP: *R* = 0.78; GC vs. RT: *R* = 0.75; RP vs. RT: *R* = 0.77), indicating that intergroup dissimilarity exceeded intragroup variation. Mantel tests revealed a nonsignificant correlation (*p* = 0.667) between host phylogenetic distance (mitochondrial cytb-based) and microbial Bray–Curtis dissimilarity (Supplementary Figure S2).

### 3.3 Bacterial community composition at different taxa levels

**Figure 4:**
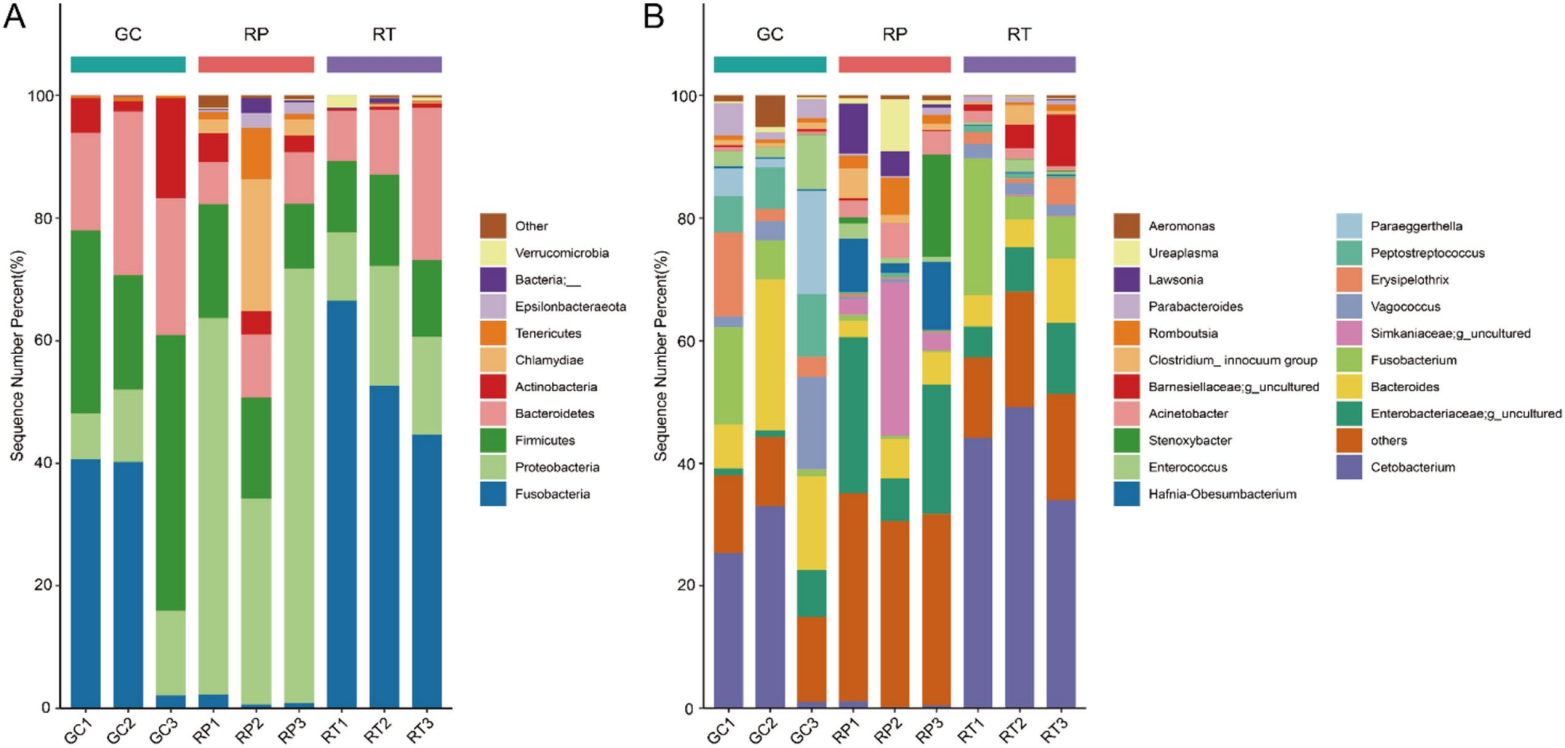
Taxonomic classifications of three groups at the **(A)** phylum and **(B)** genus levels. X-axis: three groups. Y-axis: Percent abundance of phylum and genus.

At the phylum level, dominant phyla were defined as >30% relative abundance, with subdominant phyla at >10%. The dominant phylum of GC was identified as Firmicutes (31.16% ± 13.29%), followed by Fusobacteria (27.67% ± 22.13%), Bacteroidetes (21.63% ± 5.41%), and Proteobacteria (11.01% ± 3.18%) as subdominant phyla. The dominant phylum of RP was Proteobacteria (42.30% ± 19.39%), with Firmicutes (14.75% ± 4.15%) representing the secondary dominant phylum. RT showed Fusobacteria dominance (54.70% ± 4.15%). The secondary dominant phyla were Proteobacteria (15.57% ± 4.17%), Bacteroidetes (14.49% ± 8.99%), and Firmicutes (13.00% ± 1.70%). (Figure 4A and Supplementary Table S1). LSD *post hoc* tests revealed significant intergroup differences in the dominant phylum of the three snakes at the phylum level. Fusobacteria abundance was significantly higher in RT versus RP, while Proteobacteria showed significantly greater abundance in RP compared to GC and RT (Supplementary Table S1).

At the genus level, dominant genera were classified at >10% abundance, with subdominant genera >7%. The dominant genera of GC were identified as *Cetobacterium* (19.80% ± 16.68%) and *Bacteroides* (15.69% ± 8.72%), while the subdominant genera were *Fusobacterium* (7.77% ± 7.43%), *Peptostreptococcus* (7.61% ± 2.23%), and *Paraeggerthella* (7.58% ± 8.15%). The dominant genera of RP were Enterobacteriaceae; *g*_*uncultured* (17.88% ± 9.66%) and Simkaniaceae; *g_uncultured* (10.07% ± 12.93%), while *Hafnia*-*Obesumbacterium* (7.08% ± 9.85%) were the secondary dominant genera. The dominant genera of RT were identified as *Cetobacterium* (42.42% ± 7.72%) and *Fusobacterium* (10.99% ± 9.95%), while the secondary dominant genera were Enterobacteriaceae; *g_uncultured* (7.96%±3.35%) (Figure 4B and Supplementary Table S2).

**Figure 5:**
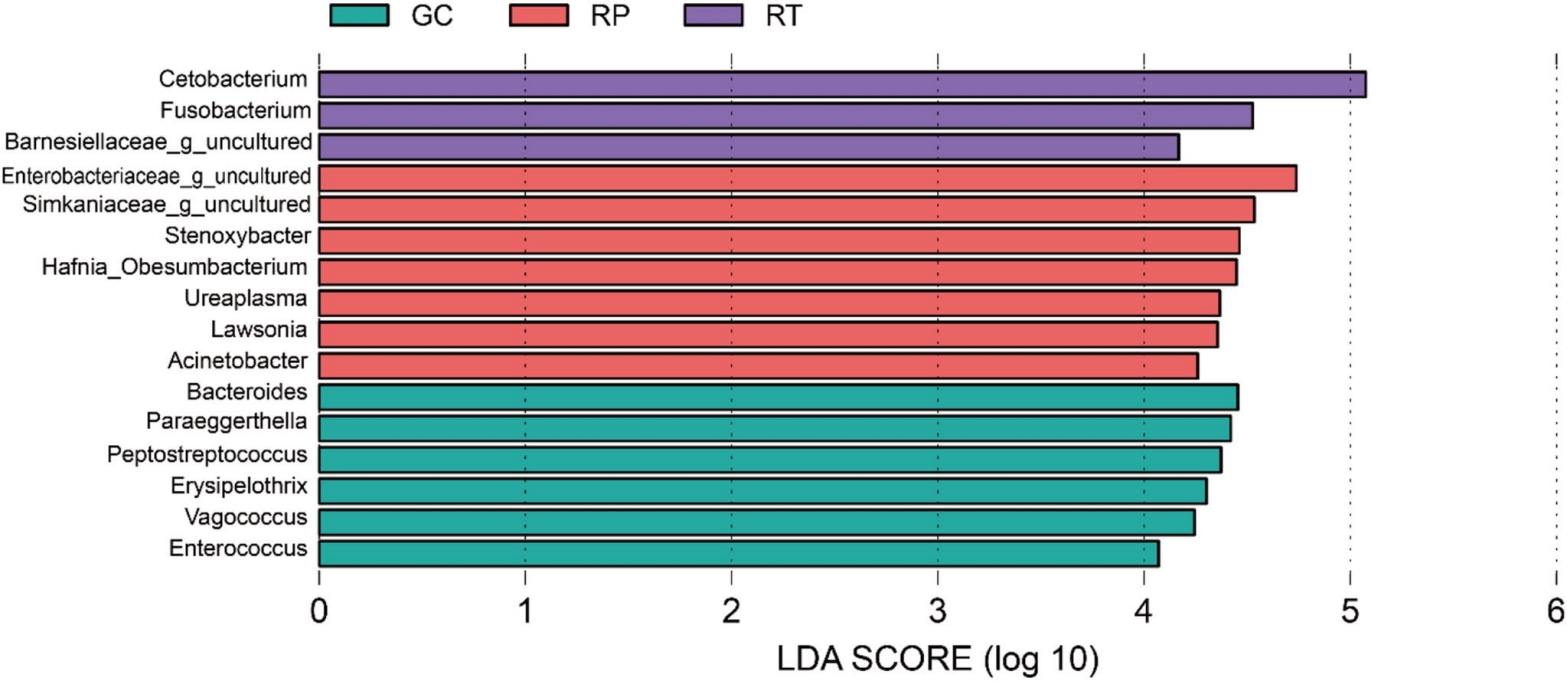
Differentially identified by linear discriminant analysis coupled with effect size (LEfSe).

The LEfSe (LDA > 4, *p* < 0.05) analysis was performed on all bacteria at the genus level. A total of 16 bacteria with a significant difference were found. Six were found in GC (*Bacteroides, Paraeggerthella, Peptostreptococcus, Vagococcus, Erysipelothrix, Enterococcus*), and seven were found in RP (Enterobacteriaceae; *g_uncultured*, Simkaniaceae; *g_uncultured*, *Hafnia_Obesumbacterium*, *Stenoxybacter*, *Lawsonia*, *Ureaplasma*, *Acinetobacter*), and three were found in RT (*Cetobacterium*, *Fusobacterium,* Barnesiellaceae; *g_uncultured*) (Figure 5). Besides that, *Cetobacterium* is one of 16 top taxa abundant in RT. This finding is also consistent with the changes in *Cetobacterium* mentioned above.

### 3.4 Co-occurrence network

**Figure 6:**
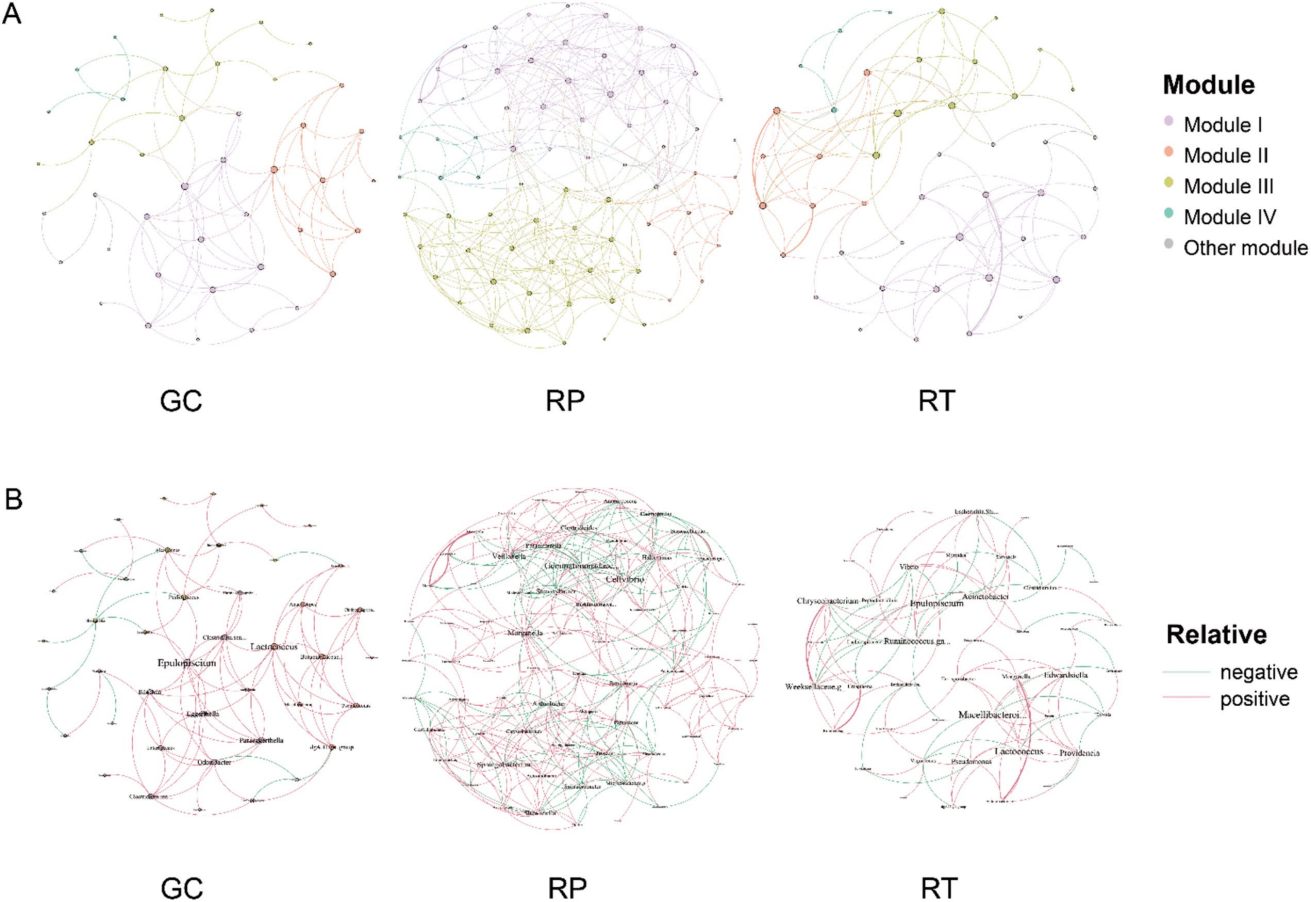
Symbiotic networks of gut bacterial communities: the gut bacterial networks of three snake species are shown: GC, RP, and RT. the networks are based on Spearman’s correlation between enriched taxa (relative abundance of genera >1%), indicating strong connectivity (|r| > 0.6) and significant (*p* < 0.05) correlation (*p*< 0.05) correlation. Node and name size are proportional to the number (degree) of links. Network of frog gut samples colored according to modularity **(A)**, with red and green lines indicating positive and negative correlations, respectively **(B)**.

To characterize microbial interactions, we constructed genus-level co-occurrence networks based on Spearman correlation analysis (*ρ* > |0.6|, *p* < 0.05) for taxa exceeding 1% relative abundance, with node degree filtering (>2 connections). The network’s basic topological properties were then analyzed (Figure 6A and Supplementary Table S3). Among the three snake species, RP exhibited the most complex network, with the highest number of nodes (73), total links (386), negative links (28.76%), and average degree (10.575). This indicates extensive interactions and a highly competitive microbial community. In contrast, GC displayed the smallest network with the fewest nodes, links, and negative associations, suggesting a predominantly collaborative microbial structure. Modular analysis revealed similar modularity across all networks, with GC showing the highest modularity (0.516) and clustering coefficient (0.631), indicating a tendency to form localized cooperative groups.

There were variations in the key taxa between networks (Figure 6B). In GC, *Epulopiscium* (p_Firmicutes), *Lactococcus* (p_Firmicutes), and *Odoribacter* (p_Bacteroidetes) were identified as critical taxa. For RP, key genera included *Cellvibrio* (p_Proteobacteria), Gemmatimonadaceae.*g_uncultured* (p_Gemmatimonadetes), and *Sphingobacterium* (p_Bacteroidetes). In RT, *Epulopiscium* (p_Firmicutes), *Lactococcus* (p_Firmicutes), and *Macellibacteroides* (p_Bacteroidetes) played an important role. Proteobacteria and Firmicutes were the two dominant clades in the symbiotic network of GC (37.7%; 35.5%), RP (39.1%; 25.6%), and RT (35.5%; 31.1%). Notably, these dominant taxa mainly interact positively with other genera, highlighting their integrating role in the network.

### 3.5 Differences in the function of a bacterial community prediction

**Figure 7:**
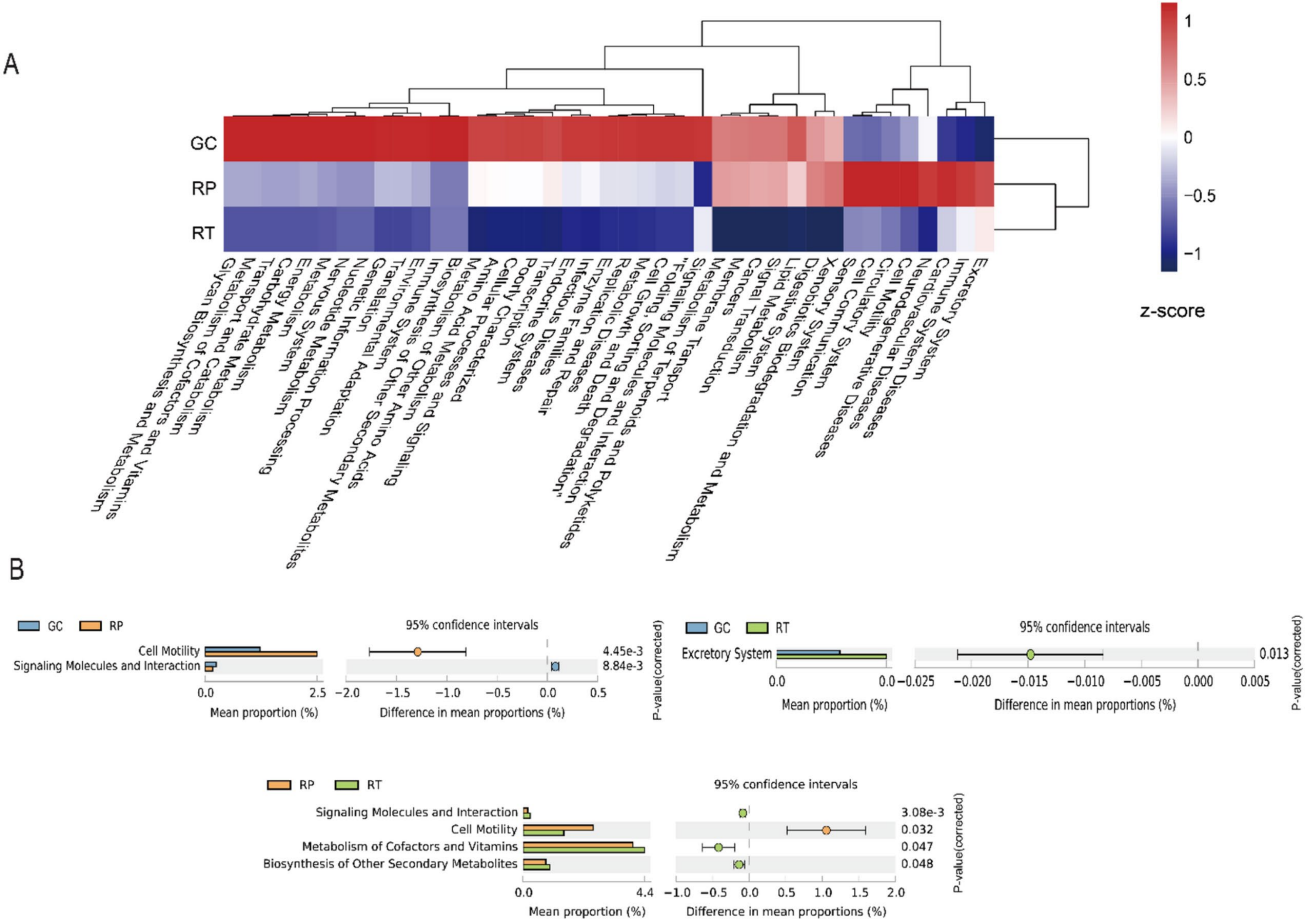
Predicted function pathways analysis among three snakes groups. **(A)** Heatmap of predicted function pathways. **(B)** Differences among groups of predicted function pathways were compared through software STAMP and two-side Welch’s *t*-test (*p* < 0.05).

The heatmap analysis demonstrated higher relative abundances of core metabolic pathways (carbohydrate metabolism, energy metabolism, and metabolism of cofactors and vitamins) and genetic information processing (replication, repair, and translation) in the GC group compared with RP and RT groups (Figure 7A). STAMP analysis further identified distinct functional specializations: (1) RP showed elevated cell motility compared to GC and RT; (2) GC and RT groups displayed higher abundances of signaling molecules and interaction pathways than RP; (3) The RT group demonstrated significantly increased excretory system activity relative to GC, alongside upregulated metabolism of cofactors and vitamins and biosynthesis of secondary metabolites compared to RP (Figure 7B).

## 4 Discussion

### 4.1 The relationship between gut microbiota and dietary preference

Human evolutionary studies have revealed that long-term dietary shifts not only shape the adaptive evolution of the host genome but also drive the co-evolution of gut microbial communities. Comparative studies across mammalian species have demonstrated that dietary patterns are a key determinant of gut microbiota composition. For instance, Bacteroides enterotypes exhibit a strong positive correlation with the intake of animal proteins, amino acids, and saturated fats, whereas the abundance of Firmicutes and Proteobacteria is positively associated with dietary fiber consumption (Ley et al., 2008b). Importantly, experimental evidence from other vertebrates underscores that diet exerts a more profound influence on gut microbiota structure than host phylogeny or environmental factors (Colston and Jackson, 2016). Fusobacterium is enriched in captive lizards that feed earthworms, but the abundance of *Elizabethkingia*, *Halomonas*, *Morganella*, and *Salmonella* in captive lizards that feed loaches is higher (Jiang et al., 2017).

Previous studies have identified Proteobacteria as a common bacterial phylum in various snake species, including *Agkistrodon piscivorus* (Colston et al., 2015), *Crotalus horridus* (McLaughlin et al., 2015), *Rhabdophis subminiatus* (Tang et al., 2019), *Laticauda laticaudata*, *Trimeresurus flavomaculatus*, and *Boiga dendrophila* (Smith et al., 2021), as well as *Ptyas mucosus* (Wei et al., 2023) all contained Proteobacteria phyla. The dominant bacterial phyla of the three snake species include Proteobacteria and Firmicutes (Figure 1B). Notably, Fusobacteria (54%) was dominant in both RT and GC. RP exhibited a significantly higher abundance of Proteobacteria (42%) compared to GC and RT. This likely relates to their primarily invertebrate-based diet. Indeed, significant differences in microbial diversity and community structure exist between vertebrate and invertebrate hosts (Brooks et al., 2016). Insect hosts typically harbor microbiota dominated by Proteobacteria, a pattern also observed in insectivorous lizards such as *Japalura sensu lato*, *Shinisaurus crocodilurus*, *Phrynocephalus vlangalii*, and *Takydromus septentrionalis*. Conversely, omnivorous species (e.g., *Liolaemus parvus*) and herbivores (e.g., *Phymaturus williamsi*) exhibit lower proportions of Proteobacteria in their intestines (Tian et al., 2022). Proteobacteria not only enhance cellulolytic activity, degrade various aromatic compounds, and promote nutrient absorption but also play a dual role in intestinal immunity (Reid et al., 2011). Under healthy conditions, they contribute to anti-infection and anti-inflammatory processes; however, excessive proliferation can disrupt gut microbial homeostasis, serving as a key marker of dysbiosis (Shin et al., 2015).

Fusobacteria is commonly found in scavengers, being particularly enriched in the hindgut of omnivores and carnivores. For instance, Fusobacteria constitute about 21% of the gut microbiota in black vultures and 31% in turkey vultures. This relationship is mutually beneficial, as Fusobacteria thrive in a protein-rich, anaerobic environment while the scavenger host benefits from the bacterial degradation of carrion to obtain nutrients (Roggenbuck et al., 2014). Fusobacteria are also frequently isolated from infected reptiles (Stevens et al., 2009). Research on the digestive microbiota of *Alligator mississippiensis* has revealed that Fusobacteria is a core phylum in its gut ecosystem. This bacterial group may contribute to the development of digestive organs and nutrient absorption and is involved in amino acid metabolism. However, it might interfere with the host’s efficiency in protein degradation (Keenan et al., 2013). In carnivorous species such as tigers and lions, Fusobacteria is distinctly more prevalent than in omnivores (e.g., gibbons, golden monkeys, chimpanzees, Assam macaques, giant pandas, black bears, and red pandas), suggesting a close association with a specialized meat-based diet (Chen et al., 2018). Fusobacteria are prevalent in GC and RT. We hypothesize that wild snakes, which typically experience long intervals between meals and consume prey much larger than themselves, may have adapted to support bacteria specialized in carrion digestion. This prolonged digestive process, sometimes lasting several days, could favor the presence of Fusobacteria, which aid in breaking down carrion and efficiently extracting nutrients for the host.

In GC and RT, the dominant bacterial genera were *Cetobacterium* and *Fusobacterium*, while *Bacteroides* was a dominant genus found only in GC. In contrast, Enterobacteriaceae; *g_uncultured,* and Simkaniaceae; *g_uncultured* were dominant only in RP. The diets of GC and RT appear more similar, as reflected by the similarities in their dominant gut genera compared to RP. To further investigate how diet differences impact gut microbiota composition among these three snake species, we performed LEfSe analysis to identify genera with higher relative abundance. The results indicated that *Cetobacterium*, Enterobacteriaceae; *g_uncultured*, Simkaniaceae; *g_uncultured*, and *Bacteroides* were primarily responsible for the observed microbial differences among the three snakes. The Enterobacteriaceae are facultatively anaerobic bacteria capable of both respiratory and fermentative metabolism. Previous studies have reported Enterobacteriaceae in the guts of various earthworm species, where they function as a highly active group within the gut microbiota, particularly in environments lacking added sugars (Meier et al., 2018). Enterobacteriaceae are highly active fermenters in the earthworm gut, utilizing glucose-derived carbon, which suggests that they may also metabolize mucus and plant-derived sugars during gut transit (Wust et al., 2011). Previous studies on snakes have identified Enterobacteriaceae as abundant in species such as *Lycodon rufozonatus* (Shang et al., 2023), *Hydrophis curtus,* and *H. cyanocinctus* (Zhong et al., 2022). Simkaniaceae, a member of the phylum Chlamydiae, was first recognized as a dominant bacterial group in studies of snake microbiota. We hypothesize that Simkaniaceae may play a role in regulating host physiology and the immune response, although its exact ecological functions require further investigation.

*Bacteroides* is strictly anaerobic, with roles in lactose fermentation and carbohydrate metabolism. These metabolic capabilities allow them to contribute significantly to the digestive processes of both herbivores and carnivores, engaging in multiple metabolic pathways (Turnbaugh et al., 2006). *Sceloporus aeneus*, *S. bicanthalis*, *S. spinosus,* and *S. grammicus* harbor mainly *Bacteroides* and *Parabacteroides* (Hernandez et al., 2024). Dietary habits strongly influence the structure of gut microbial communities; for instance, a carbohydrate-rich diet promotes the growth of *Prevotella*, whereas a protein- and fat-rich diet supports the proliferation of *Bacteroides* (Wu et al., 2011). This relationship is further substantiated by the observed increase in Bacteroides within the gut microbiota of animals that consume protein- and fat-rich diets, which reflects the interplay between protein fermentation and carbohydrate metabolism (David et al., 2014). Notably, *Bacteroides* not only serves as a dominant genus in GC but also shows a high relative abundance in both RP and RT, collectively confirming the carnivorous nature of all three snake species. Interestingly, previous studies have shown that Fusobacteria are commonly found in the intestines of omnivorous and carnivorous freshwater fish, where they play roles in biofilm formation, digestive organ development, and nutrient absorption (Larsen et al., 2014), and function as a dominant phylum in fish gut microbiota (Chen et al., 2018; Eichmiller et al., 2016). In our study, RT exhibited a notable abundance of Fusobacteria, especially *Cetobacterium*, mirroring findings in fish studies. Cetobacterium is the dominant genus in the gut microbiota of most fish species, such as common carp (*Cyprinus carpio*) (van Kessel et al., 2011), and Nile tilapia (*Oreochromis niloticus*) (Ray et al., 2017). *Cetobacterium* metabolites, particularly short-chain fatty acids (SCFAs) like acetate, propionate, and butyrate, along with vitamin B12, have been shown to benefit fish health (Bhute et al., 2020; Wang et al., 2021; Xie et al., 2021).

### 4.2 Cluster perspective: the effect of dietary preference on the gut microbiota

Although dietary diversity is generally assumed to correlate positively with gut microbial *α*-diversity, our data reveal that the obligate feeder RP exhibits significantly higher Observed_species, Chao1, and ACE indices than the vertebrate-feeding GC and RT. This unexpected pattern likely reflects the metabolic demands associated with specialized feeding strategies, particularly the degradation of complex chitin in invertebrate exoskeletons. Statistical validation via PERMANOVA (*R*^2^ = 0.33, *p* < 0.001) confirmed significant microbial differentiation among species. Notably, this evolutionary convergence parallels findings in myrmecophagous mammals (Delsuc et al., 2014) and amphibians (Brunetti et al., 2023), suggesting that dietary specialization promotes cross-species functional convergence through either elevated *α*-diversity or conserved enrichment of core degradative taxa (e.g., chitinolytic communities).

Host genetics, dietary divergence, and physiological traits all influence gut microbiota composition (Benson et al., 2010; Moeller et al., 2020; Spor et al., 2011). However, our study demonstrates that dietary divergence plays a dominant role in snakes. Take the genus *Elaphe* as an example: despite the high morphological and habitat similarity between *E. schrenckii* and *E. anomala* and their historical classification as conspecific subspecies, significant genus-level microbiota differences exist between them (Lu et al., 2019). This pattern of dietary influence extends even to phylogenetically distant species, as hosts with similar diets exhibit convergent microbial communities (Brooks et al., 2016). The dendrogram based on ASVs demonstrated that RT and GC shared more similar microbiota compared to RT and its conspecific RP. Moreover, the Mantel test indicated no significant correlation between host phylogenetic distance (based on the *cytb* gene) and microbiota dissimilarity (*p* = 0.667). This is consistent with our PCoA results, where PCoA2 effectively distinguishes RT from the other species due to its elevated abundance of *Cetobacterium*, a genus associated with aquatic prey consumption. In contrast, GC clusters along PCoA1 are driven by its higher proportion of Bacteroides, which reflects its vertebrate-based diet. Interestingly, *Cetobacterium* is also a dominant genus in GC, potentially due to the following factors: (1) All samples were collected during the rainy season, a period of increased amphibian activity that may have led GC to consume more frogs. (2) Prey size in the GC and RT groups was significantly larger than in the RP group. Their intermittent feeding strategy may favor symbiont colonization, as frequent feeding-digestion cycles tend to select for rapidly growing taxa (e.g., Enterobacteriaceae), whereas intermittent feeding supports the proliferation of symbiotic bacteria such as *Cetobacterium*. In summary, the specialized feeding behavior of RP not only enhances its gut microbial α-diversity but also drives functional specialization within its microbiome, leading to significant compositional differences compared to closely related species that predominantly consume vertebrates. The mechanism by which dietary specialization promotes the convergent evolution of microbial functions appears to be conserved across different taxonomic groups, as evidenced in myrmecophagous mammals and amphibians (Brunetti et al., 2023; Delsuc et al., 2014), thereby offering a novel perspective for understanding host-microbe co-evolution.

### 4.3 Diet-microbiota network associations in wild snakes

Network analysis provides new perspectives on microbial interactions (Grond et al., 2019), and differences in diet can lead to significant changes in gut microbial diversity and network structure (Ley et al., 2008a; Youngblut et al., 2019). Additionally, the study demonstrates that diet types (herbivorous, omnivorous, and carnivorous) play a dominant role in the gastrointestinal microbiome networks of Nematoda, Arthropoda, and Chordata, with their impact being significantly greater than host phylogeny (Ma and Shi, 2024). Network stability can be quantitatively assessed using modularity and the negative edge ratio (Hernandez et al., 2021; Newman, 2006). High modularity is positively correlated with ecological network stability (Newman, 2006), whereas a low negative edge ratio reduces competitive interference, facilitating host adaptation to environmental fluctuations (Grilli et al., 2016). Although not in the same genus, GC and RT exhibited highly stable microbial networks potentially attributable to their overlapping feeding strategies (vertebrate prey species). The observed dietary diversification appears to promote synergistic inter-colony interactions (91.11% positive links in GC vs. 76.1% in RT) and may contribute to enhanced modular structures (modularity > 0.5). Conversely, RP’s specialized diet (earthworms vs. fireflies exclusively) coincided with heightened inter-colony competition (28.76% negative interactions), forming a complex network (73 nodes, 386 links) with reduced modularity (0.498) that demonstrated comparatively lower stability than GC/RT. These patterns may indicate that dietary diversity supports colony stability, while dietary similarity could drive functional convergence of microbial communities across phylogenetically divergent hosts - a phenomenon analogous to that reported in migratory birds (Wang et al., 2022). Our findings align with the hypothesis that the host diet selects key species to establish stable functional taxa (Youngblut et al., 2019), though internal resource competition within these taxa might modulate network resilience (Feng et al., 2017). Notably, GC and RT shared core hub taxa *Epulopiscium* (Firmicutes) and *Lactococcus* (Firmicutes), which could maintain host homeostasis through putative nutrient decomposition (Parata et al., 2020) and metabolic regulation (Li et al., 2023). The RP network displayed the smallest diameter (6) and relatively low density (0.147), suggesting a configuration where centralized microbial connectivity might be particularly vulnerable to keystone node perturbations (e.g., *Cellvibrio*, *Sphingobacterium*). *Cellvibrio* and Sphingobacteriaceae are jointly involved in initial chitinolysis to provide energy to the host (Wieczorek et al., 2019), potentially supplying energy substrates for the host. Monitoring abundance fluctuations in these core microbiota (e.g., *Cellvibrio*, *Sphingobacterium*) may provide insights for ecological risk assessment in RP systems.

### 4.4 The function composition of the gut microbiota to diet

KEGG pathway analysis indicated that RT exhibits enhanced capabilities in cofactor/vitamin metabolism and secondary metabolite biosynthesis, which may reflect its adaptation to high-protein diets. For example, the enrichment of *Cetobacterium somerae* in its gut could support host hemoglobin production via vitamin B12 synthesis (Qi et al., 2023), although this function requires validation through the host erythrocyte parameters. In contrast, the relatively low abundance of signal molecule interaction pathways in RP might indicate a need for a stable gut microbial community. Moreover, the slightly higher abundances of amino acid metabolism and environmental adaptation functions observed in the gut microbiota of GC likely reflect the metabolic demands associated with processing the diverse components of terrestrial prey. Similar patterns have been observed in other systems. For instance, the functional divergence between the gut microbial communities of baleen and toothed whales (Sanders et al., 2015) suggests that substrate types transmitted through food chains (e.g., krill as the primary food for baleen whales, which in turn feed on phytoplankton) may drive metabolic specialization. In snakes, differences in prey origin, such as the ingestion of insect exoskeletons rich in chitin, may influence microbial functions through the food chain dynamics. Notably, the host genetic background may influence microbial functions by modulating the gut’s physical environment (Figure 1C). For instance, the enrichment of cell motility functions in RP might be linked to its frequent feeding and digestive cycles. Similarly, the persistence of carnivorous gut characteristics during the dietary transition in giant pandas suggests that host genes constrain microbial functions (Hu et al., 2017; Nie et al., 2019). It is plausible to infer that the food chain plays a significant role in shaping gut microbial composition, much like the evolutionary transition of the giant panda’s carnivorous gut into a specialized bamboo-based system—a process reflecting the long-term co-evolution of dietary habits and species.

Limited by the elusive behavior and capture difficulty of wild snakes, we obtained only three biological replicates per species (*n* = 3). While this small sample size aligns with precedents (Colston et al., 2015; Li et al., 2021; Tang et al., 2019; Zhang et al., 2024), it may constrain statistical power and generalizability. Furthermore, by focusing exclusively on carnivorous snakes (GC, RT, RP), our findings may lack broader relevance compared to studies across dietary guilds. Nevertheless, our data suggest a predictive link between microbial networks and snake dietary specificity, though experimental validation is required to confirm causality. Additionally, using PICRUSt2 on 16S rRNA data infers only potential metabolic capabilities rather than actual activities or host–microbe interactions. For example, although RT showed predicted enrichment in excretory system functions (e.g., the urea cycle), validation through serum urea level measurements or assessments of intestinal ammonia concentrations is required. Future studies should integrate multi-omics approaches, such as metagenomics and metabolomics, to elucidate the quantitative relationships between dietary components and microbial network parameters, thereby unraveling the link between dietary specificity and microbial adaptation. Such an approach would further validate the functionality of the gut microbiota and explore its ecological roles.

## 5 Conclusion

This study analyzed the gut microbiota of three snake species, revealing distinct microbial compositions and network structures. The invertebrate-feeding RP exhibited higher alpha diversity, although its less stable microbial network was more susceptible to prey scarcity. Network analysis highlighted differences in key taxa: *Epulopiscium* and *Lactococcus* were identified as keystone taxa in GC and RT; their presence and abundance underscore the critical role of dietary preferences in shaping gut microbial communities. *Cellvibrio* and *Sphingobacterium* in RP could serve as monitoring indicators. Functional predictions further reflected adaptations of the gut microbiota to distinct ecological niches, suggesting that dietary habits influence microbial assemblages through food chain dynamics.

## Data Availability

The original contributions presented in the study are publicly available. This data can be found here: https://www.ncbi.nlm.nih.gov/, accession number: PRJNA1191237.

## Footnotes

^1^ https://www.bioincloud.tech

^2^ http://www.omicshare.com/tools
