# Supplementary material for: Dietary preferences affect the gut microbiota of three snake species (Squamata: Colubridae)
Source: Front Microbiol. 2025 May 21;16:1559646. doi: 10.3389/fmicb.2025.1559646 (PMC12136495; doi:10.3389/fmicb.2025.1559646)
Supplement: Supplementary file 1 [file Data_Sheet_1.pdf]

# *Supplementary Material*

## 1. Supplementary Figures

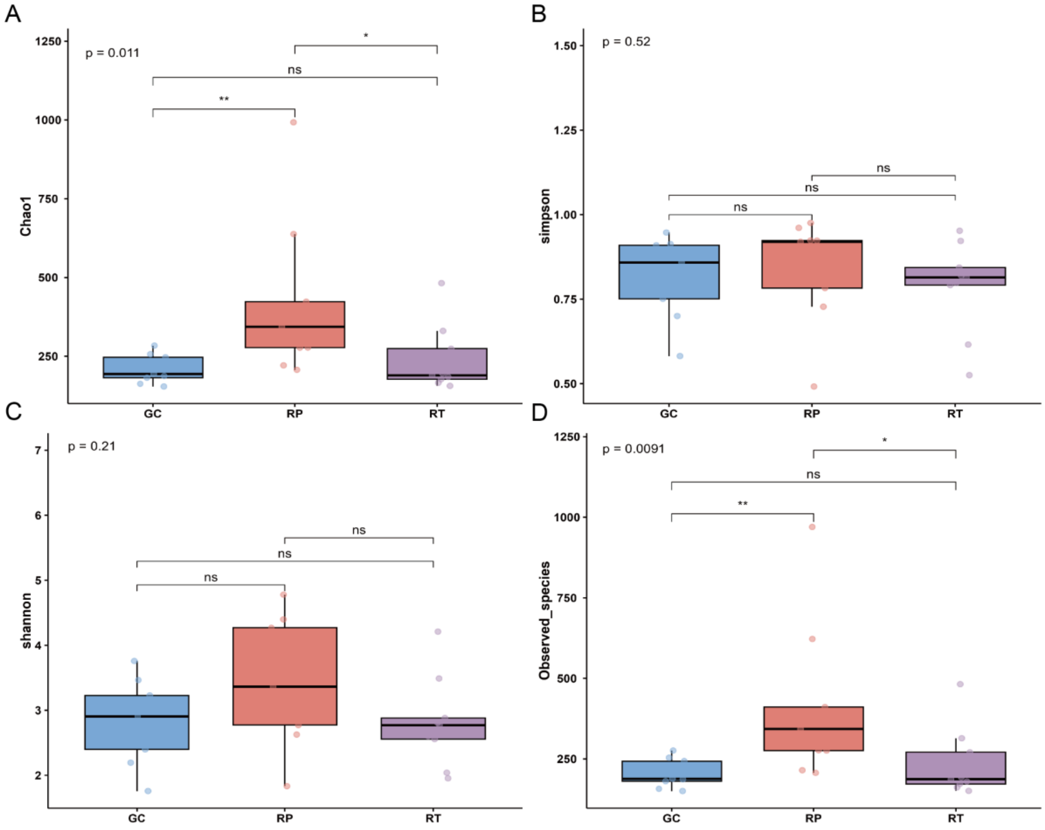


**Supplementary Fig. S1** Comparison of alpha diversities of gut microbiota among the three snake species. Chao1 measures species richness(A); Simpson and Shannon measure species diversity(B;C); Observed_species measures species number distribution(D). Different color represents the different groups, “*” Represents the significant differing Shannon index between the two groups (p-values of less than 0.05)
