# Supplementary material for: Dietary preferences affect the gut microbiota of three snake species (Squamata: Colubridae)
Source: Front Microbiol. 2025 May 21;16:1559646. doi: 10.3389/fmicb.2025.1559646 (PMC12136495; doi:10.3389/fmicb.2025.1559646)
Supplement: Supplementary file 2 [file Data_Sheet_2.pdf]

Supplementary Material

1 Supplementary Figures

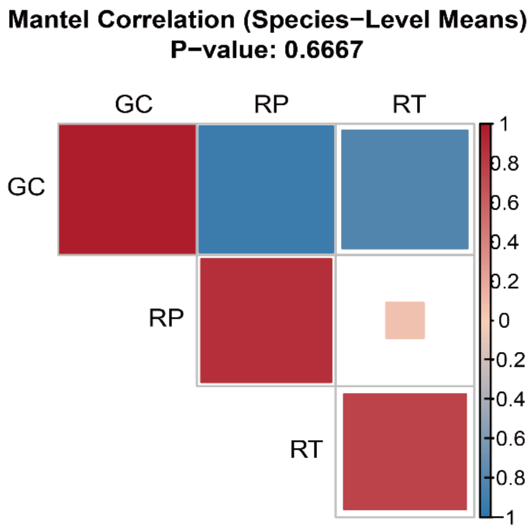

Supplementary Fig. S2 Heatmap shows the correlation between host phylogenetic distance and microbial community distance, assessed by Mantel test.
